# Contribution of White Matter Fiber Bundle Damage to Language Change After Surgery for Temporal Lobe Epilepsy

**DOI:** 10.1212/WNL.0000000000206862

**Published:** 2023-04-11

**Authors:** Lawrence Peter Binding, Debayan Dasgupta, Peter Neal Taylor, Pamela Jane Thompson, Aidan G. O'Keeffe, Jane de Tisi, Andrew William McEvoy, Anna Miserocchi, Gavin P. Winston, John S. Duncan, Sjoerd B. Vos

**Affiliations:** From the Department of Computer Science (L.P.B., S.B.V.), Centre for Medical Image Computing, Department of Clinical and Experimental Epilepsy (L.B.P., D.D., P.N.T., P.J.T., J.d.T., A.W.M., A.M., G.P.W., J.S.D.), UCL Queen Square Institute of Neurology, and Neuroradiological Academic Unit (S.B.V.), UCL Queen Square Institute of Neurology, University College London; Victor Horsley Department of Neurosurgery (D.D., A.W.M., A.M.), and Department of Neuropsychology (P.J.T.), National Hospital for Neurology and Neurosurgery, Queen Square, London; CNNP Lab (P.N.T.), Interdisciplinary Computing and Complex BioSystems Group, School of Computing Science, Newcastle University; School of Mathematical Sciences (A.G.O.), University of Nottingham; Epilepsy Society MRI Unit (J.d.T., G.P.W., J.S.D.), Chalfont Centre for Epilepsy, Chalfont St Peter, United Kingdom; Department of Medicine (G.P.W.), Division of Neurology, Queen's University, Kingston, Canada; and Centre for Microscopy (S.B.V), Characterisation, and Analysis, The University of Western Australia, Nedlands, Australia.

## Abstract

**Background and Objectives:**

In medically refractory temporal lobe epilepsy (TLE), 30%–50% of patients experience substantial language decline after resection in the language-dominant hemisphere. In this study, we investigated the contribution of white matter fiber bundle damage to language change at 3 and 12 months after surgery.

**Methods:**

We studied 127 patients who underwent TLE surgery from 2010 to 2019. Neuropsychological testing included picture naming, semantic fluency, and phonemic verbal fluency, performed preoperatively and 3 and 12 months postoperatively. Outcome was assessed using reliable change index (RCI; clinically significant decline) and change across timepoints (postoperative scores minus preoperative scores). Functional MRI was used to determine language lateralization. The arcuate fasciculus (AF), inferior fronto-occipital fasciculus (IFOF), inferior longitudinal fasciculus, middle longitudinal fasciculus (MLF), and uncinate fasciculus were mapped using diffusion MRI probabilistic tractography. Resection masks, drawn comparing coregistered preoperative and postoperative T1 MRI scans, were used as exclusion regions on preoperative tractography to estimate the percentage of preoperative tracts transected in surgery. Chi-squared assessments evaluated the occurrence of RCI-determined language decline. Independent sample *t* tests and MM-estimator robust regressions were used to assess the impact of clinical factors and fiber transection on RCI and change outcomes, respectively.

**Results:**

Language-dominant and language-nondominant resections were treated separately for picture naming because postoperative outcomes were significantly different between these groups. In language-dominant hemisphere resections, greater surgical damage to the AF and IFOF was related to RCI decline at 3 months. Damage to the inferior frontal subfasciculus of the IFOF was related to change at 3 months. In language-nondominant hemisphere resections, increased MLF resection was associated with RCI decline at 3 months, and damage to the anterior subfasciculus was related to change at 3 months. Language-dominant and language-nondominant resections were treated as 1 cohort for semantic and phonemic fluency because there were no significant differences in postoperative decline between these groups. Postoperative seizure freedom was associated with an absence of significant language decline 12 months after surgery for semantic fluency.

**Discussion:**

We demonstrate a relationship between fiber transection and naming decline after temporal lobe resection. Individualized surgical planning to spare white matter fiber bundles could help to preserve language function after surgery.

Temporal lobe resection is an effective surgical treatment for medically refractory temporal lobe epilepsy (TLE). However, individuals undergoing language-dominant resection have a 30%–50% risk of significant postoperative decline in language-related functions.^1^ Word-finding difficulties can affect daily life.^2^ Consequently, it is important to try to minimize the impact of temporal lobe surgery on language function.

Lateralization of visual and auditory naming functional MRI (fMRI) activations in the ipsilateral temporal lobe predicts patients who will undergo a language decline.^3^ However, surgically sparing fMRI-activated cortical regions does not avoid a naming decline in 50% of individuals.^4^ Language function is dependent on a network involving multiple dispersed cortical regions.^5^ Communication between these distant cortical regions is enabled by white matter fiber bundles, which are thus essential for language function.^6^

There have been several attempts to characterizing white matter involvement in postoperative language decline. White matter is anatomically organized in fiber bundles. Research using diffusion MRI (dMRI) found that preoperative fractional anisotropy measures of the inferior longitudinal fasciculus (ILF) and inferior fronto-occipital fasciculus (IFOF) fasciculi correlated with postoperative picture and auditory naming decline, respectively.^7^ Further research has extended this association by evaluating postoperative fractional anisotropy measures that correlate with postoperative language scores.^8^ Whilst these studies correlate preoperative and postoperative scores to preoperative and postoperative diffusion metrics, they do not address the relationship between surgically induced white matter damage and postoperative language decline.

Our aim in this study was to determine the correlations between surgical damage to language-related white matter tracts and the occurrence of postoperative language decline. We investigated several language-related fiber bundles that are at risk of damage during surgery: the arcuate fasciculus (AF), uncinate fasciculus (UF), ILF, middle longitudinal fasciculus (MLF), and IFOF.^9^ The ultimate goal was to improve neurosurgical planning in each patient by avoiding these tracts and minimize the risk of language function decline; analogous to the avoidance of surgical damage to the optic radiation for preventing visual field defects.^10^

## Methods

### Participants

One hundred sixty-one consecutive patients who underwent TLE surgery at the National Hospital of Neurology and Neurosurgery, London, United Kingdom, between 2010 and 2019 were included. No patients underwent invasive language mapping, and dMRI of language bundles was not considered when planning resections. 34 patients were excluded because of the following reasons: previous neurosurgery (N = 11), incomplete data (N = 12), or bilateral language representation (N = 11). All remaining patients had a preoperative T1-weighted structural MRI; dMRI; task-based language fMRI, and a postoperative T1-weighted MRI (obtained between 3 and 12 months postoperatively).

Patients were stratified according to their language lateralization, derived from clinical reports of language fMRI and the quantitative fMRI lateralization index (LI)^11^ based on a verbal fluency task.^12^ Groups were defined by an LI > +0.2 (left hemisphere dominant), −0.2 < LI < 0.2 (bilateral), and LI < −0.2 (right hemisphere dominant). Patients were dichotomized as having surgery on the language-dominant (n = 65) or language-nondominant (n = 62) hemisphere.

### Standard Protocol Approvals, Registrations, and Patient Consents

This project was approved by London—Bloomsbury Research Ethics Committee (REC reference: 20/LO/0149; CAG number: 20/CAG/0013). Patient data were pseudoanonymized using a subject identification number that carried no information about the patient but could be referenced on a database with patient information if required. All patients had the opportunity to opt out of research. This project did not carry any risk to participants and was retrospectively conducted on clinically acquired data.

### Neuropsychology

Patients underwent the McKenna Graded Naming Test (referred to as picture naming),^13^ phonemic verbal fluency (letter S, referred to as phonemic fluency) assessment, and categorical verbal fluency (category: animals, referred to as semantic fluency) assessment.^14^ These were performed preoperatively and postoperatively at 3 and 12 months. Patients with missing data on an assessment were excluded from analysis for that assessment only. For phonemic fluency, only the letter “S” was performed because this was a presurgical screening assessment.

Change in neuropsychological performance was assessed using the reliable change index (RCI) and preoperative and postoperative changes. For picture naming, an RCI decline of ≥4 was considered a clinically significant decline as per previous research.^3^ For semantic and phonemic fluency, we used the test-retest RCIs, which were corrected for practice effects.^15^ RCI was calculated as the SD of score difference between assessment 1 and assessment 2 and multiplied by 1.645 (Z_CI_ from the normal distribution). This equated to a decline of ≥9 for semantic fluency and ≥7 for phonemic fluency being a significant decline.^16^ Language change was calculated as postoperative-preoperative scores.

### MRI Acquisition

Between 2009 and 2013,80 patients were scanned on a 3T GE Signa Excite HDx. Single-shell dMRI data were acquired using a cardiac-triggered single-shot spin-echo planar imaging sequence^14^: 1.875 × 1.875 × 2.4 mm resolution, gradient directions: 6 and 52 at b values: 0 and 1,200/mm^2^, and δ/Δ/TE = 21/29/73 ms, and a 3D T1-weighted sequence was acquired as described in Taylor et al.^17^ Task-based verbal fluency and generation^14^ gradient-echo planar T2*-weighted fMRI were acquired with 58 contiguous 2.5-mm oblique axial slices, 96 × 96 matrix reconstructed to 128 × 128 for an in-plane resolution of 1.875 × 1.875 mm (TE/TR = 25/2,500 ms).

Between 2014 and 2019, 47 patients were scanned on a 3T GE Discovery MR750. A 3D T1-weighted sequence (MPRAGE) was acquired as described in Vos et al.,^18^ and multishell dMRI data were acquired (2 mm isotropic resolution, gradient directions: 11, 8, 32, and 64 at b values: 0, 300, 700, and 2,500 s/mm^2^; ∂/Δ = 21.5/35.9 ms, and TE/TR = 74.1/7,600 ms). Task-based verbal fluency and generation^14^ gradient-echo planar T2*-weighted fMRI were acquired with 50 contiguous 2.4-mm (0.1 mm gap) slices with a 24-cm field of view, 64 × 64 matrix with an in-plane voxel size of 3.75 × 3.75 mm (TE/TR = 22/2,500 ms).

### MRI Processing

#### Diffusion Processing

dMRI data were denoised,^19^ Gibbs-unringed,^20^ corrected for signal drift,^21^ and distortion corrected using a synthesized b0 for diffusion distortion correction (Synb0-DisCo)^22^ with FSL topup.^23^ Eddy currents and movement artifacts were corrected,^24^ rotating the b vectors.^25^ In addition, bias field correction was performed in MRtrix3.^22^ Response functions for the CSF, white matter, and gray matter were estimated using Single-Shell 3-Tissue^27^ and Multi-Shell 3-Tissue^28^ CSD in MRtrix3.^22^

#### fMRI Processing

Hemispheric language lateralization was calculated using the bootstrap method of the LI toolbox implemented in SPM8^29^ on verbal fluency spmT maps, using the WFU PickAtlas' anatomical masks of the middle and inferior frontal gyrus (including the pars triangularis, orbitalis, and opercularis).^30^ LI values were calculated as follows: (LI = [L–R]/[L + R]).

#### Resection Mask

Resection masks were drawn based on previous techniques.^17^ Postoperative T1-weighted MRI were affinely registered to preoperative T1-weighted MRI. Resection masks were then manually drawn in MRtrix3 by overlaying the postoperative T1-weighted MRI on the preoperative T1-weighted MRI starting at the most anterior coronal slice of the temporal lobe and then proceeding posteriorly every 3 slices. Coronal slices were then joined by drawing in every sagittal slice. Masks were saved in preoperative T1-weighted space. Resection mask reliability and validity were assed through inter-rater reliability between 2 raters. Impact of delineation accuracy was assessed using dilated resection masks (eTables 1 and 2 in eAppendix 1, links.lww.com/WNL/C631).

#### Anatomically Targeted–Automated Tractography

Details on tractography reconstruction can be seen in eAppendix 2 (links.lww.com/WNL/C632), and details on cortical terminations have been listed in eTable 3 (eAppendix 2).

Change in fiber bundles from preoperative to estimated postoperative was calculated as the percentage difference using the following formula: ([postoperative−preoperative] ÷ preoperative) × 100.

### Statistical Analysis

Statistical analysis was performed to assess the relationship between RCI decline and the following clinical features: fMRI LI, age at epilepsy onset, epilepsy duration during surgery, seizure freedom at 12 months (ILAE outcome 1), and resection volume. In addition, the relationship between RCI decline and the following fiber bundles were analyzed: AF, IFOF, ILF, MLF, and UF.

We used a χ^2^ test to assess whether there was a difference in RCI decline between patients with language-dominant resections and those with language-nondominant resections.

To assess feature differences between those with RCI decline and nondecline in those with language-dominant resections and those with language-nondominant resections, we used independent sample *t* test with false discovery rate (FDR) to control for multiple comparisons. This was used to identify features that could have a linear relationship to language change.

We used a robust linear regression to determine whether there was subfascicle specialization within the fiber bundles significant at the RCI *t* test analysis and show whether there was a linear relationship or a cutoff point at which performance drops. We used language change (postoperative-preoperative scores) as the dependent variable. We picked the MM-estimator^31^ regression algorithm for its ability in controlling for outliers, performing similarly to ordinary least squares on uncontaminated data.^32^ Variables entered into the model as fixed effects were based on features that showed significance in the 3-month or 12-month independent sample *t* test analysis (Dominant vs Nondominant Hemisphere section). Fiber bundles significant in the *t* test analysis were split into their respective subfasciculi. Confounding effects (fMRI LI and resection volume) were included in all models. Features were normalized before inclusion in the model by shifting the mean to 0 and scaling to have an SD of 1. All features were entered into the regression, and the robust final prediction error (RFPE)^31^ was calculated. Features were removed one by one to minimize the RFPE (indicating a better model). To assess the impact of outlier handling in the robust estimator, we repeated these regressions using a second robust regression method, the talwar algorithm, which also has demonstrated performance on our sample size (eAppendix 3, links.lww.com/WNL/C633).

### Sensitivity Analysis

To assess whether results were dependent on a combination of more limited temporal lesionectomies and anterior temporal lobe resection (ATLR), we performed the same analysis on a subcohort of ATLR patients. A full comparison of subgroups is listed in eTable4 (eAppendix 4, links.lww.com/WNL/C634) and visualized in eFigures 1–2.

To assess whether the results of this study could be modeled across both 3-month and 12-month decline, we applied the final models of this study in a generalized mixed-effect model. The results of this analysis are summarized in eTable 5 (eAppendix 5, links.lww.com/WNL/C635), and pitfalls are discussed and visualized in eFigures 3–4.

### Data Availability

Anonymized data that these results were based on and were not published within this article will be made available on request from any qualified investigator.

## Results

A summary of significant features to language assessments is given in Table 1. Only significant findings are reported, and detailed statistics of nonsignificant findings are summarized in eTable 6–11 (eAppendix 6, links.lww.com/WNL/C636).

**Table 1:**
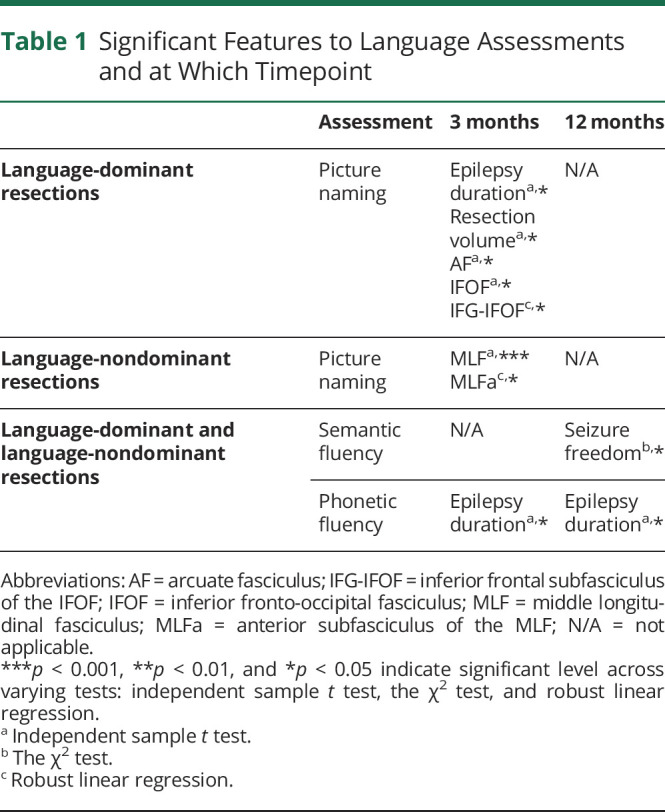
Significant Features to Language Assessments and at Which Timepoint

### Descriptive Statistics

Demographic information is summarized in Table 2.

**Table 2:**
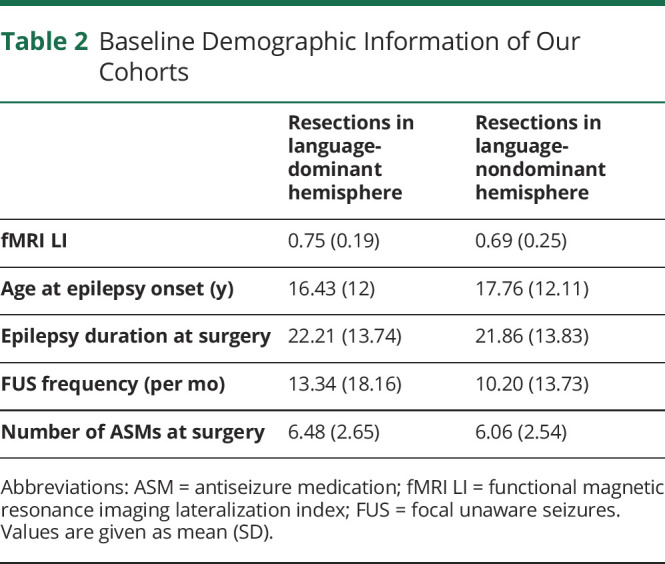
Baseline Demographic Information of Our Cohorts

The 65 language-dominant hemisphere patients (33 female) comprised the following: 61 with left-language lateralization and left resection; 4 with right-language lateralization and right resection. Fifty-four patients underwent ATLR, and 11 underwent a more limited lesionectomy. Pathology in this group included the following: hippocampal sclerosis (N = 36), cavernoma (CAV; N = 6), dysembryoplastic neuroepithelial tumor (DNT; N = 10), dual pathology (N = 6), and other (N = 7). There were several patients with missing scores for picture naming at 3 months (N = 8) and 12 months (N = 20), semantic fluency at 3 months (N = 7) and 12 months (N = 19), and phonemic fluency at 3 months (N = 7) and 12 months (N = 19). These patients were excluded from these assessments only.

The 62 language-nondominant hemisphere patients (38 female) comprised the following: 57 with left-language lateralization and right resection; 5 with right-language lateralization and left resection. 57 patients underwent ATLR, and 5 underwent a more limited lesionectomy. Pathology for this group included the following: HS (N = 32), CAV (N = 4), DNT (N = 7), dual pathology (N = 5), and other (N = 12). There were several patients with missing scores for picture naming at 3 months (N = 8) and 12 months (N = 17), semantic fluency at 3 months (N = 4) and 12 months (N = 15), and phonemic fluency at 3 months (N = 4) and 12 months (N = 15). These patients were excluded from these assessments only.

### Language Performance

#### Hemispheric Dominance and Performance

Preoperative and postoperative language scores are summarized in Table 3. Cross-sectional analysis was performed to identify whether there were significant differences in scores between language-dominant and language-nondominant groups. A χ^2^ test of independence was used to assess group differences of those that did have RCI decline at 3 and 12 months between language-dominant and language-nondominant patients.

**Table 3:**
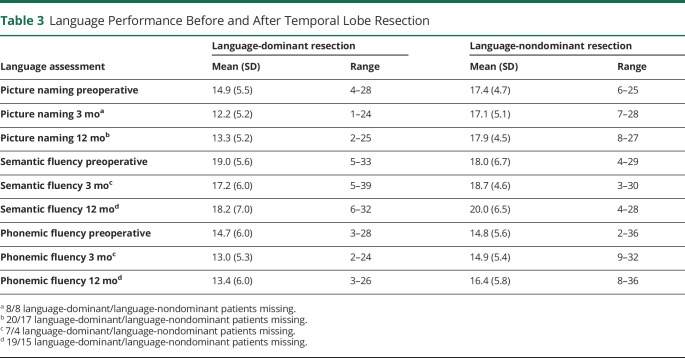
Language Performance Before and After Temporal Lobe Resection

For picture naming, patients with language-dominant resections had lower scores across all 3 timepoints compared with those with language-nondominant resections (Table 3). Furthermore, a chi-squared assessment showed significant differences between the number of patients that had declined at 3 months on language-dominant resections (19/57, 33.3% of patients) compared with language-nondominant resections (5/54, 9.3%) (χ(1) = 9.483, *p* = 0.002, odds = 4.900, 95% CI 1.677–14.139) and at 12 months with significantly higher language-dominant (12/45, 26.7%) than language-nondominant (2/45, 4.5%) resections causing RCI decline (χ(1) = 8.459, *p* = 0.004, odds = 7.818, 95% CI 1.636–37.360). This demonstrates there were clinically significant different outcomes between language-dominant and language-nondominant hemisphere resections. As such, our remaining analysis will use separate language-dominant and language-nondominant groups to identify clinically significant differences per group.

For semantic fluency, surgery in language-dominant patients was associated with a drop in performance at 3 months and a slight improvement at 12 months but not reaching preoperative levels (Table 3). By contrast, semantic fluency scores were higher after surgery to nondominant temporal lobes at both 3 and 12 months. A χ^2^ test, however, of those who had RCI decline showed that there were no significant differences in the language-dominant patients (6/58, 10.3%) compared with language-nondominant patients (3/58, 5.2%) at 3 and 12 months (language-dominant patients = 5/46, 10.9% vs language-nondominant = 2/47, 4.3%). This suggests there are no clinically significant differences in semantic fluency outcome between language-dominant and language-nondominant resections. Our remaining analysis will combine dominant and nondominant resections into 1 group.

For phonemic fluency, language-nondominant groups had higher preoperative scores than the dominant group (Table 3). However, a chi-squared assessment of those that had RCI decline showed that there were no significant differences between the language-dominant (8/58, 13.8% of patients) and language-nondominant resections (3/58, 5.2% of patients) at 3 and 12 months (language-dominant = 6/46, 13% of patients vs nondominant = 5/47, 10.6% of patients). Our remaining analysis will combine language-dominant and language-nondominant resections in 1 group.

### Differences in Resections and Change in Language

#### Scanner Effect on Features

An independent sample *t* test showed there was a significant difference between scanner type and AF resection (*p* = 0.001, d = 0.587, 95% CI 0.225–0.947). Consequently, the AF was harmonized across scanners.^33^

#### Dominant vs Nondominant Hemisphere

To assess feature differences between language-dominant and language-nondominant patients, we used an independent sample *t* test at an alpha level of 0.05 with FDR correction. Resection volume was 29.0% greater on the nondominant (mean = 34.8 mL ± SD = 9.8 mL) than on the dominant hemisphere resections (27.0 ± 9.8 mL): *p* < 0.001, Cohen d (d) = 0.784, 95% CI 0.421–1.144. IFOF resection was 51.0% greater on the nondominant (46.8% ± 31.8%) than on the dominant hemisphere resections (31.0% ± 33.5%): *p* = 0.007, d = 0.484, 95% CI 0.130–0.836. ILF resection was 39.9% greater on the nondominant (82.6% ± 16.3%) than on the dominant hemisphere resection (59.0% ± 28.7%): *p* < 0.001, d = 1.004, 95% CI −0.633 to 1.372. MLF resection was 338.7% greater on the dominant (28.2% ± 13.5%) than on the nondominant hemisphere resections (8.3% ± 15.3%): *p* < 0.001, d = 1.375, 95% CI 0.985–1.760.

#### RCI Group-Level Feature Differences

To assess feature differences between those with and without RCI decline, we used an independent sample *t* test at an alpha level of 0.05 with FDR correction. For picture naming on the language-dominant hemisphere at 3 months: epilepsy duration was 30% greater for those with RCI decline (27.0 ± 15.9 years) than those without RCI decline (18.9 ± 11.9 years): *p* = 0.033, d *=* −0.613, 95% CI −1.173 to −0.048. Resection volume was 28.5% greater for those with (31.9 ± 9.7 mL) than for those without RCI decline (24.8 ± 10.3 mL): *p* = 0.016, d = −0.697, 95% CI −1.260 to −0.128. AF resection as 218.5% greater for those with (5.9% ± 7.7%) than for those without RCI decline (2.7% ± 3.6%): *p* = 0.032, d = −0.619, 95% CI −1.179 to −0.053. IFOF resection was 91.9% greater for those with (44.7% ± 38.3%) than for those without RCI decline (23.3% ± 27.9%): *p* = 0.019, d = −0.676, 95% CI −1.238 to −0.109. There were no significant differences at 12 months.

For picture naming on the language-nondominant hemisphere at 3 months, MLF resection was 486.2% greater for those with RCI decline (31.2% ± 39.8%) than for those without RCI decline (5.3% ± 6.9%): *p* < 0.001, d = −2.009, 95% CI −2.998 to −1.003. There were no significant differences at 12 months.

For semantic fluency at 3 months and 12 months, there were no significant differences. For phonemic fluency at 3 months postoperatively, epilepsy duration at operation was 42.5% greater for those with RCI decline (29.6 ± 14.4 years) than for those without RCI decline (20.8 ± 13.3 years): *p* = 0.040, d = −0.658, 95% CI −1.283 to −0.029. This same relationship was observed at 12 months, where epilepsy duration at operation was 46.9% greater for those with RCI decline (28.6 ± 15.11 years) than for those without RCI decline (19.5 ± 13.2 years): *p* = 0.037, d = −0.679, 95% CI −1.314 to −0.040.

#### Seizure Freedom and Language Outcome

To assess whether there was a significant difference in those with and those without RCI decline and 1-year seizure freedom, we used a chi-squared assessment.

For picture naming, there were no significant differences at 3 or 12 months on the language-dominant or language-nondominant hemisphere. For semantic fluency, there were no significant differences at 3 months. At 12 months, there was a significant difference between those who were seizure-free without RCI decline (58.1%) compared with those with RCI decline (14.3%): *p* = 0.025, odds = 0.120, 95% CI 0.01–1.040. For phonemic fluency, there were no significant differences at 3 or 12 months.

#### Seizure Freedom and Resection Volume

An independent sample *t* test for both language-dominant and language-nondominant resections showed there was no significant difference between resection volume and seizure freedom at 1 year.

### Correlation of Subfascicles and 3-Month or 12-Month Neuropsychology Change

To assess whether there was a linear relationship between features and neuropsychology score change from preoperative to 3 or 12 months postoperatively (postoperative-preoperative score), we used a robust least squares regression. Features assessed were based on significant group differences between those with and without RCI decline (Dominant vs Nondominant Hemisphere section). Fiber bundles were segmented into subfasciculi according to previous research. Confounds (fMRI LI and resection volume) were added to each model.

#### Picture Naming

##### Language-Dominant Hemisphere

The IFOF was segmented into 3^34^ and the AF into 2 subfasciculi.^35^ Resection of the AF's ventral subfasciculus was significantly different between scanner types (*p* = 0.006, d = 0.724, 95% CI 0.210–1.234) and was harmonized^33^ to remove scanner effect.

**Figure 1:**
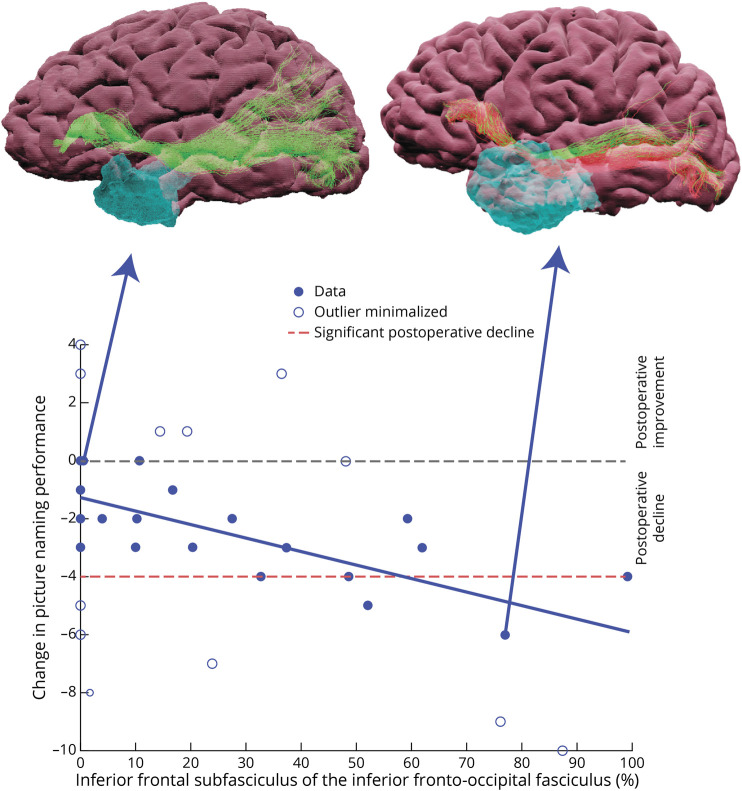
Scatter Plot of Language-Dominant Picture Naming Change at 3 Months and the Percentage of IFG-IFOF Resection Patient outliers were identified by a robust linear regression with the open circles indicating outliers where their weighting in the model was reduced. The dotted horizontal red line indicates the level of significant decline indicated by the reliable change index. Example patient resections are shown as 3D visualizations showing remaining fibers (green) and resected (red) due to resection cavity (blue). IFG-IFOF =inferior frontal subfasciculus of the inferior fronto-occipital fasciculus.

For picture naming at 3 months, the best model (Table 4; RFPE = 0.1895, χ^2^(1,39) = 4.906, *p* = 0.027, adjusted R^2^ = 0.137) included the following: confounds (fMRI LI [*p* = 0.392], total resection volume [*p* = 0.650]) and surgical damage to the inferior frontal subfasciculus of the IFOF (IFG-IFOF; *p* = 0.033, β = −1.417, 95% CI 0.163–2.671) (Figure 1). This translates to IFG-IFOF damage resulting in an increased risk of picture naming decline, explaining 13.7% of decline. This model outperformed a confounds-only model (see Table 4 for full details). An example of a patient with the IFOF spared is shown in Figure 3A. The best model was marginally different in the typical ATLR subgroup of patients, with the IFG-IFOF maintaining significance (see eTable 10, eAppendix 5).

**Table 4:**
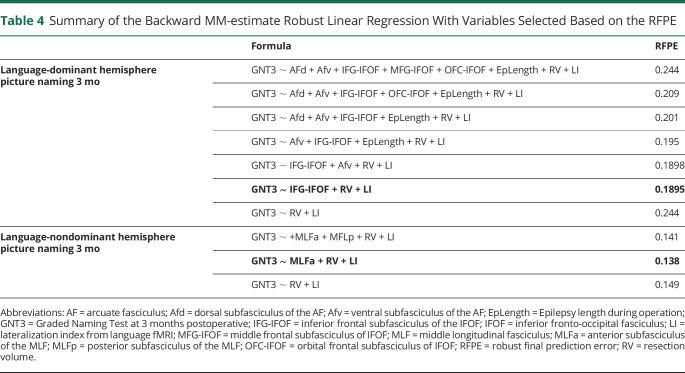
Summary of the Backward MM-estimate Robust Linear Regression With Variables Selected Based on the RFPE

##### Language-Nondominant Hemisphere

**Figure 2:**
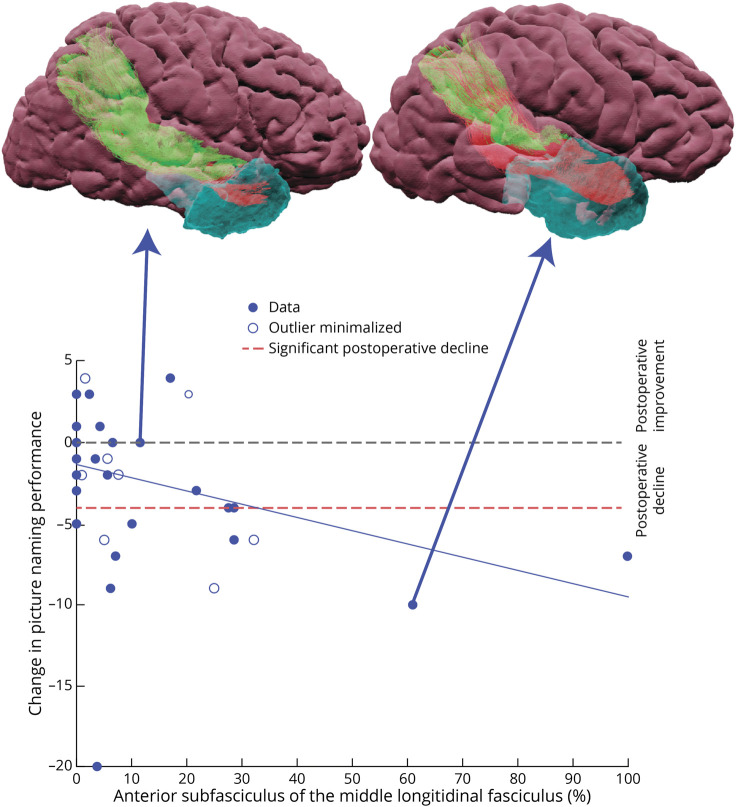
Scatter Plot of Language-Nondominant Picture Naming Score Change at 3 Months and the Percentage of the MLFa Resection Patient outliers were identified by a robust linear regression with the open circles indicating outliers where their weighting in the model was reduced. The dotted horizontal red line indicates the level of significant decline indicated by the reliable change index. Example patient resections are shown as 3D visualizations showing remaining fibers (green) and resected (red) due to resection cavity (blue). MLFa = anterior subfasciculus of the middle longitudinal fasciculus.

**Figure 3:**
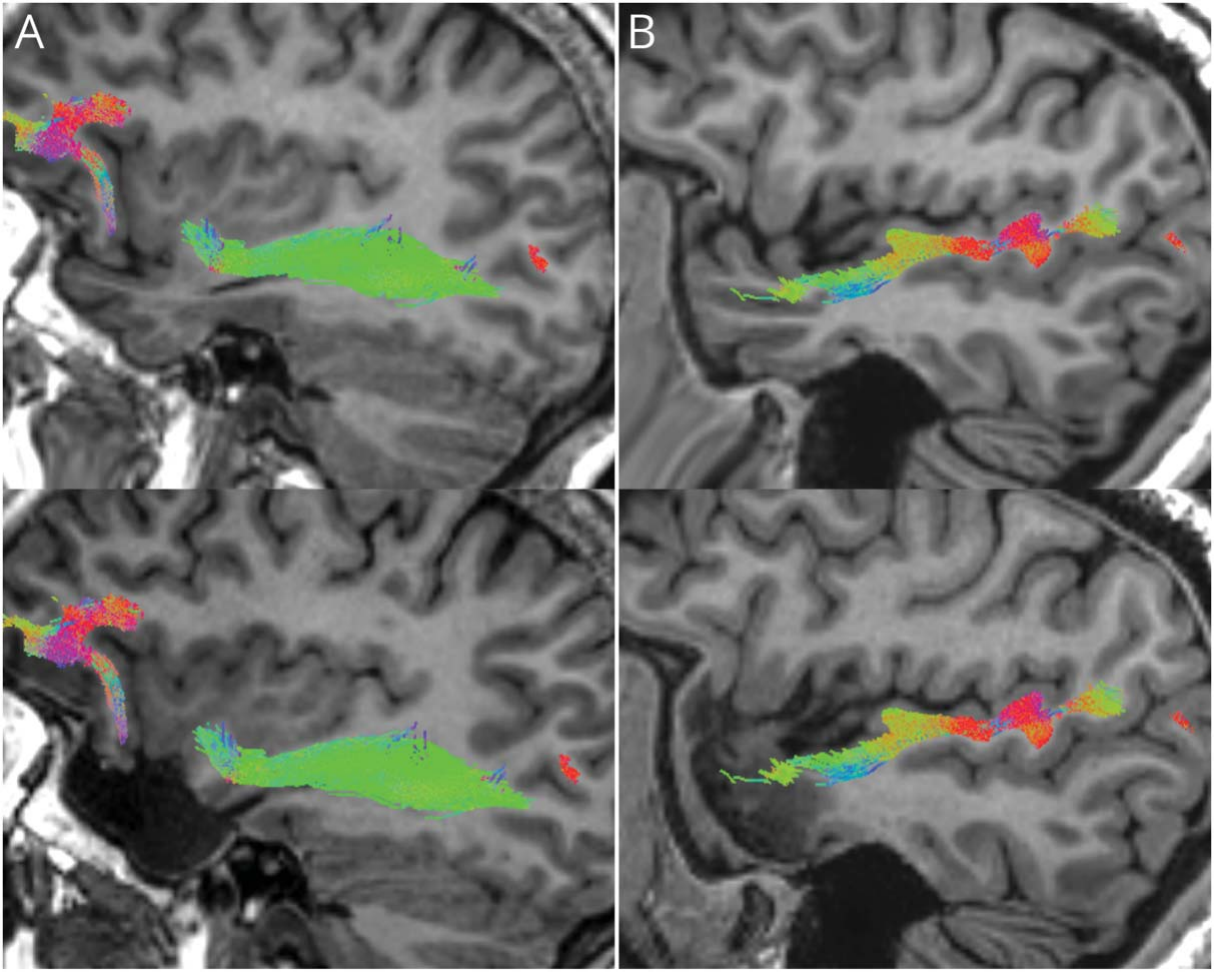
Sagittal Representation of a Patient With the Inferior Fronto-occipital Fasciculus (A) and Middle Longitudinal Fasciculus (B) Spared in Left Anterior Temporal Lobe Resection For each bundle, preoperative (top) and postoperative (bottom) T1-weighted images are shown with tracts overlaid.

The MLF was segmented into 2 subfasciculi.^36^ For picture naming at 3 months, the best model (Table 4; RFPE = 0.138, χ^2^(1,49) = 6.601, *p* = 0.010, adjusted R^2^ = 0.073) included the following: confounds (fMRI LI [*p* = 0.650], total resection volume [*p* = 0.707]), and surgical damage to the anterior subfasciculus of the MLF (MLFa; *p* = 0.013, β = −0.351, 95% CI −0.618 to −0.083; Figure 2). Practically, this translates to MLFa damage, resulting in an increased risk of picture naming decline, explaining 7.3% of decline. This model outperformed a confounds-only model (see Table 4 for full details). An example of a patient with the MLF spared is shown in Figure 3B. Analysis of the typical ATLR subgroup of patients included same features in the best model but no overall significance (see eTable 10, eAppendix 5).

#### Semantic and Phonemic Fluency

There were no significant preoperative or postoperative features associated with semantic or phonemic fluency outcome.

## Discussion

Previous research has implicated white matter fiber bundles in preoperative or postoperative language function in TLE surgery,^37^ albeit with limited translational capability for surgical targeting to prevent language decline after surgery. Using resection masks and preoperative tractography, we document a direct relationship between picture naming and fiber bundles transection, which is clinically implementable for future surgery.

Typically, patients are split into language-dominant and language-nondominant resections when assessing the risk of language decline. We demonstrated significantly different outcomes for picture naming between these groups, supporting previous literature.^38^ However, there was no significant difference in semantic and phonemic fluency outcome between language-dominant and nondominant resections. Thus, analyses of picture naming outcome split patients into language-dominant and language-nondominant resections, whereas both groups were combined for semantic and phonemic fluency analyses.

### Picture Naming—Language-Dominant Resection

At 3 months, we showed that there is a significant difference between IFOF resection, AF resection, epilepsy age at onset, and resection volume between those with and without RCI decline. These were not significant at 12 months. Modeling picture naming change as a linear combination of these features, the IFG-IFOF was significantly correlated with outcome, with greater damage being associated with worse language outcome. In the ATLR-only subgroup, we demonstrated the same IFOF subfasciculus correlated with language change (eTable 1, eAppendix 1, links.lww.com/WNL/C631).

Our findings support that preservation of the IFOF is related to postoperative picture naming function.^7^ The IFOF has been implicated in picture naming ability, although there is no consensus on the exact function of IFOF.^5^ Solely, the IFG-IFOF was correlated with naming decline. This suggests a functional specialization within the IFOF, which may account for inconsistencies in the literature that measured the bundle as an unspecific whole.

The AF interconnects the superior, middle, and inferior temporal gyri to the frontal lobe.^5^ The middle and inferior temporal gyri are both involved in semantic storage.^5^ Our results highlight the role of the AF in relaying semantic information to the frontal lobe for picture naming ability.

Resection volume is a combination of white and gray matter resections. This suggests that both gray matter and white matter resections may play a role in picture naming decline at 3 months—reinforcing picture naming as a multifaceted function involving dispersed cortical regions requiring structural connections.^6^

Earlier onset of TLE is associated with atypical functional language representation.^39^ Hence, there could be efficient functional reorganization (i.e., away from the epileptogenic zone) with earlier onset. Future research confirming this would open the possibility of targeted therapies to promote reorganization away from the anterior temporal lobe before surgery.^40^

### Picture Naming—Language-Nondominant Resection

At 3 months, there were significant group differences in MLF resection between those with and without RCI decline. Modeling picture naming change as a linear combination of predictive features, resection of MLFa connections were significantly correlated with significant decline. In the ATLR-only subgroup, this model remained the best but lost overall significance (eTable 1, eAppendix 1, links.lww.com/WNL/C631).

The MLF terminations (superior temporal gyrus and temporal pole to the parietal lobe) are important for language function.^5^ We find evidence for a role of the MLF in picture naming function. MLFa extensions are implicated in retrieving auditory information consolidated in the temporal lobe.^41^ There is evidence in the literature that the superior temporal gyrus in TLE is involved in semantic function.^5^ Future research should try and delineate if any fMRI-activated regions in TLE overlap with the MLF in picture naming to confirm our finding.

### Semantic Fluency—Language-Dominant and Language-Nondominant Resections

Continued seizures 12 months after language-dominant and language-nondominant resections were associated with semantic fluency impairment. We infer that ongoing seizure activity is related to the continued dysfunction of functional networks.

### Phonemic Fluency—Language-Dominant and Language-Nondominant Resections

Longer duration of epilepsy was significantly related to an RCI decline of phonemic fluency at 3 months.

Epilepsy duration is an indirect measure of cumulative seizure burden. Previous research has shown high performance on phonemic fluency is contingent on a highly connected network of dispersed cortical regions across the frontal and parietal lobes.^43^ The strength of connectivity in the frontal and parietal regions could be negatively affected by long-term seizure burden^44^ and thus lead to poor performance postoperatively. Future research should aim to clarify whether clinical factors directly affect frontal lobe connectivity.

### Clinical Impact

The language network is complex and widespread, and recovery of healthy function after surgery can occur with gray and white matter plasticity, facilitating functional reorganization.^45^ Surgical damage to both gray and white matter has been associated with postoperative naming decline, but this has not been translated into clinical practice.^37^ In this study, we present findings that can be used in clinical settings to mitigate some of the risks of temporal lobe surgery to language function.

Typically, a standard ATLR in the language-dominant temporal lobes involves a complete dissection of the temporal UF and anterior-temporal extensions of AF, MLF, and ILF, with resection of the anterior 2–3 cm of the superior temporal gyrus, the anterior hippocampus, and amygdala. Middle and inferior temporal gyri resection extends 4–5 cm posterior to the pole, aiming to spare the posterior temporal cortex, including the fusiform gyrus. The IFOF runs along the boundary of the resection margin, which explains the high variability in the extent of resection. Adapting dominant temporal lobe surgery to avoid IFOF while reducing the lateral neocortical resection may mitigate postoperative picture naming impairment. In the nondominant temporal lobe, greater proportions of superior temporal gyrus and lateral neocortex are typically resected. Our results suggest that preserving the MLFa will mitigate adverse effects on picture naming function.

Sparing the IFOF and MLF during surgery to help preserve some language function could be possible with smaller resections because we showed resection size was not related to postoperative seizure freedom. However, there was individual variation in white matter fiber bundles anatomy. As such, to increase the specificity of surgery in preserving language, an intraoperative display overlaying the tractographic representations could be used. We have established this technique to be beneficial to preserving vision in the case of the optic radiation.^10^ We aim to implement this technique by displaying the IFOF, MLF, and the optic radiation^10^ for optimal neurocognitive outcomes.

### Research Evaluation

All patients included in this study had surgery performed by the same 2 surgeons. This had the benefit of ensuring there was a consistent surgical approach for all cases; however, replication studies may improve the generalizability of our findings to other centers.

Several steps were taken to ensure the accuracy of our methods. For tractography: (1) a region-of-interest (ROI)-to-ROI seeding method was used, which has been shown to be highly accurate^46^; (2) probabilistic tractography was chosen for its high sensitivity; (3) tractography was performed in both directions, flipping ROIs to ensure that there was no bias in the direction of tractography and resulting in twice as many streamlines in the main stem of the subfasciculus; and (4) an automatic pruning method was used to remove spurious tracts, ensuring the main component of the fasciculus remained. These steps increased the replicability of our results.

The use of manually drawn resection masks to estimate postoperative tractography has the benefit of the rater being able to visually estimate for brain shift but may introduce human error and image registration issues. Additional analyses were performed to investigate these issues and showed minimal impact (eAppendix 2). Furthermore, some subfasciculi were not reconstructed in some patients, which resulted in reduced cohort sizes for the subfasciculi evaluations. Although this could be rectified by tracking each subfasciculus independently, this introduces new biases.

We used the percentage change between preoperative and postoperative streamline count to yield a proxy of resection damage to tracts, and we did not account for microstructural diffusion metrics. Preoperative microstructural measures within tracts have been shown to correlate with performance.^8^ Variability shown in the relationship between resection damage and language decline (Figures 1 and 2) in these patients could be due to a preexisting dysfunction of this fiber bundle. Alternatively, this could be related to plasticity potential or successful functional reorganization. Future work should explore whether any of these factors further improve the model's accuracy in helping to prevent language decline from surgical white matter damage and to balance this with potential effects on the chance of postoperative seizure freedom.

Our results suggest that white matter fiber bundle damage correlates with adverse effects on language function, demonstrating that greater damage to the IFG-IFOF in language-dominant resections and MLFa damage in language-nondominant resections are associated with poorer postoperative picture naming performance. We hope this work will lead to reducing language decline after temporal lobe resection by planning and navigating surgery to avoid these fiber bundles. In parallel, it is important to evaluate whether there is any impact on seizure outcome.

## Glossary

AF: arcuate fasciculus
Afd: dorsal subfasciculus of the AF
Afv: ventral subfasciculus of the AF
ASM: antiseizure medication
CAV: cavernoma
dMRI: diffusion MRI
DNT: dysembryoplastic neuroepithelial tumor
EpLength: epilepsy length during operation
fMRI: functional MRI
FUS: focal unaware seizures
GNT3: Graded Naming Test at 3 months postoperative
IFG-IFOF: inferior frontal subfasciculus of the IFOF
IFOF: inferior fronto-occipital fasciculus
LI: lateralization index from language fMRI
MFG-IFOF: middle frontal subfasciculus of IFOF
MLF: middle longitudinal fasciculus
MLFa: anterior subfasciculus of the MLF
MLFp: posterior subfasciculus of the MLF
N/A: not applicable
OFC-IFOF: orbital frontal subfasciculus of IFOF
RCI: reliable change index
RFPE: robust final prediction error
RV: resection volume
TLE: temporal lobe epilepsy
UF: uncinate fasciculus
